# Enzymes enabling the biosynthesis of various C_20_ polyunsaturated fatty acids in a sea urchin *Hemicentrotus pulcherrimus*

**DOI:** 10.1098/rsob.240170

**Published:** 2025-01-22

**Authors:** Yingying Peng, Yutaka Haga, Naoki Kabeya

**Affiliations:** ^1^ Graduate School of Marine Science and Technology, Tokyo University of Marine Science and Technology, 4-5-7 Konan, Minato-ku, Tokyo 108-8477, Japan; ^2^ Department of Marine Biosciences, Tokyo University of Marine Science and Technology, 4-5-7 Konan, Minato-ku, Tokyo 108-8477, Japan

**Keywords:** desaturase, elongase, non-methylene-interrupted fatty acids, polyunsaturated fatty acids

## Abstract

Sea urchins, integral to marine ecosystems and valued as a delicacy in Asia and Europe, contain physiologically important long-chain (>C_20_) polyunsaturated fatty acids (PUFA) in their gonads, including arachidonic acid (ARA, 20:4n-6), eicosapentaenoic acid (EPA, 20:5n-3) and unusual non-methylene-interrupted fatty acids (NMI-FA) such as 20:2^Δ5,11^. Although these fatty acids may partially be derived from their diet, such as macroalgae, the present study on *Hemicentrotus pulcherrimus* has uncovered multiple genes encoding enzymes involved in long-chain PUFA biosynthesis. Specifically, 3 fatty acid desaturases (FadsA, FadsC1 and FadsC2) and 13 elongation of very-long-chain fatty acids proteins (Elovl-like, Elovl1/7-like, Elovl2/5-like, Elovl4-like, Elovl8-like and Elovl6-like A–H) were identified in their genome and transcriptomes. Functional analysis showed that FadsA and FadsC2 function as a Δ5 desaturase and a Δ8 desaturase, respectively, enabling the conversion of 18:2n-6 and 18:3n-3 into ARA and EPA, respectively, along with Elovl, particularly Elovl6-like C. Elovl6-like C demonstrates elongase activity towards both C_18_ PUFA and monounsaturated fatty acids. Consequently, FadsA and Elovl6-like C enable the synthesis of several NMI-FA, including 20:2^Δ5,11^ and 20:3^Δ5,11,14^, from C_18_ precursors. This indicates that *H. pulcherrimus* can endogenously synthesize a wide variety of C_20_ PUFA and NMI-FA, highlighting active biosynthesis pathways within sea urchins.

**Figure:**
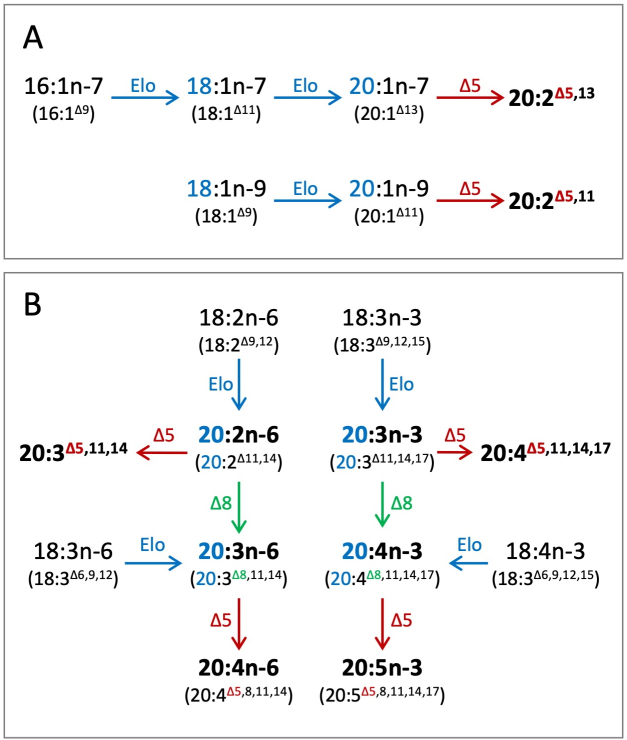

## 1. Introduction

Long-chain (≥C_20_) polyunsaturated fatty acids (LC-PUFA) are important components of cell membranes in animals and play a crucial role in maintaining their structural and functional integrity [1]. In particular, eicosapentaenoic acid (EPA, 20:5n-3) and docosahexaenoic acid (DHA, 22:6n-3) play beneficial roles in the prevention of various human diseases, including cardiovascular disease, inflammatory diseases like rheumatoid arthritis, Crohn’s disease and ulcerative colitis, as well as types of cancer such as colorectal, breast and prostate cancers. They also ensure normal cognitive and visual function during early development [2]. All deuterostomes including vertebrates and echinoderms are incapable of de novo polyunsaturated fatty acids (PUFA) biosynthesis from non-PUFA precursors due to the absence of specific enzymes [3], and thus require pre-formed C_18_ PUFA in their diets to synthesize LC-PUFA endogenously. In most eukaryotes, two types of enzymes, named front-end desaturases (Fads) and elongation of very-long-chain fatty acids proteins (Elovl), are required to synthesize LC-PUFA from C_18_ PUFA (electronic supplementary material, figure S1). The former introduces a new unsaturation (C=C double bond) between the pre-existing one and the carboxy-terminus in the fatty acyl chain, and the latter are rate-limiting enzymes catalysing the initial condensation step of fatty acid elongation to add a C_2_ unit. Elovl can further be divided into two major subgroups, according to their sequences and overall substrate preferences on either saturated/monounsaturated fatty acids (S/MUFA) or PUFA, which have been determined in vertebrates [4]. Fads also exhibit different regioselectivities to add a double bond at a specific carbon in the fatty acyl chain. Indeed, their substrate specificities and complement of genes encoding those enzymes vary among different organisms, leading to remarkable diversity in the series of LC-PUFA that can be endogenously synthesized in specific animals. The ever-increasing availability of genomic and transcriptomic information in non-model organisms has also driven the elucidation of LC-PUFA biosynthetic capacity in a variety of animals, including invertebrate species [5].

Echinoderms, a group of marine invertebrates, include extant species such as sea urchins (Echinoidea), sea cucumbers (Holothuroidea), sea stars (Asteroidea), brittle stars (Ophiuroidea) and sea lilies (Crinoidea). Numerous studies have investigated the fatty acid compositions of echinoderms, partly due to the particular commercial importance of sea urchins and sea cucumbers, which are cherished delicacies in countries across Asia, the Mediterranean and the Western Hemisphere [6,7]. Echinoderms are also of considerable ecological importance, being low trophic-level organisms with widespread distribution, ranging from intertidal to abyssal zones [8]. However, studies on the endogenous enzymes involved in LC-PUFA biosynthesis have been rather limited. Existing research includes the functional characterization of a desaturase and an elongase in the sea cucumber *Apostichopus japonicus* [9,10], and three desaturases in the sea urchin *Paracentrotus lividus* [11]. Although no functional analyses have been carried out, several studies have reported the molecular cloning of desaturases and elongases in the sea urchin *Strongylocentrotus intermedius* [12,13], as well as their regulation [14–18]. Moreover, genome-wide identification of elongases has been undertaken across several echinoderm species [19].

Fatty acid analysis has shown that commercially important sea urchins contain various PUFA, notably arachidonic acid (ARA, 20:4n-6) and EPA [8]. Although sea urchins primarily consume macroalgae which contain low amounts of LC-PUFA such as ARA and EPA, it appears these LC-PUFA might be accumulated from their diets [20,21]. However, a previous study on the sea urchin *Paracentrotus lividus* has demonstrated that they possess three Fads (FadsA, FadsC1 and FadsC2), which suggest a capacity for endogenous LC-PUFA biosynthesis [11]. Evidence from feeding trials has shown that certain sea urchins such as *P. lividus* and *Strongylocentrotus droebachiensis* were able to survive and grow normally on diets free of LC-PUFA, typically land vegetables [22–24]. This raises questions about whether they possess a complete gene set required for the full biosynthetic pathway of LC-PUFA including the physiologically important ARA, EPA and DHA from their C_18_ PUFA precursors. In addition, functional analysis of FadsA from *P. lividus* revealed its capability to synthesize unusual PUFA named non-methylene-interrupted fatty acids (NMI-FAs) from C_20_ PUFA substrates by introducing a double bond at the Δ5 position in the carbon chain. These NMI-FAs, such as 20:3^Δ5,11,14^ and 20:4^Δ5,11,14,17^, are indeed commonly detected in several Echinodermata species including sea urchins *Anthocidaris crassispina*, *S. intermedius*, *S. nudus*, *Hemicentrotus pulcherrimus* and *Pseudocentrotus depressus* [8,25], a sea star *Asterias vulgaris* [8,26] and a brittle star *Ophiura sarsi* [27]. Two other NMI-FAs (20:2^Δ5,11^ and 20:2^Δ5,13^) are also prevalent in sea urchins, with NMI-FA levels in *S. droebachiensis*, *S. intermedius* and *P. depressus* comparable to EPA levels [8,25,28]. The physiological roles of these NMI-FAs remain unclear, but their relatively high levels suggest they may play significant roles in echinoderms.

In the present study, we comprehensively isolated a series of *fads*- and *elovl*-like sequences from the transcriptomic and genomic databases of the sea urchin *H. pulcherrimus*. Subsequently, sequence and phylogenetic analyses were conducted to determine the orthology of these genes with those found in vertebrates. Furthermore, the obtained sequences were heterologously expressed in yeast for functional analysis to determine the biosynthetic capabilities of LC-PUFA in *H. pulcherrimus*.

## 2. Material and methods

### 2.1. Extraction of total RNA and cDNA synthesis

*H. pulcherrimus* (weight 11.6 g, female) used in the present study was randomly collected from intertidal areas in Tateyama Station, The Field Science Centre, Tokyo University of Marine Science and Technology (34.9763, 139.7699, Tateyama, Chiba, Japan) on 6 May 2022. Subsequently, total RNA was extracted from their gonad and intestine using TRIzol (Thermo Fisher Scientific, Waltham, MA) following the manufacturer’s recommendations. Complementary DNA (cDNA) was synthesized from 5 µg of the total RNA using a SuperScript IV Reverse Transcriptase Kit (Thermo Fisher Scientific) following the manufacturer’s recommendations.

### 2.2. *In silico* isolation of *elovl*- and *fads*-like sequences from *H. pulcherrimus* genome and transcriptome

The *elovl*- and *fads*-like sequences of *H. pulcherrimus* were comprehensively retrieved from their genome and/or transcriptome assemblies (NCBI WGS project no. BEXV01, TSA project no. IACU01) by *tblastn* using several known Elovl and Fads sequences, respectively, as queries. Three *fads*- and 13 *elovl*-like sequences were identified by this search as possible enzyme genes involved in LC-PUFA biosynthesis. Based on those putative sequences, primer sets for the full-length open reading frame (ORF) cloning were designed with the corresponding restriction enzyme site to ligate the amplified ORF into the yeast expression vector pYES2 (Thermo Fisher Scientific). All primer sequences used in the present study are listed in electronic supplementary material, table S1.

### 2.3. Phylogenetic analyses of sea urchin Fads and Elovl

The *elovl*-like sequences from Echinodermata species were retrieved from NCBI nr and TSA databases using *blastp* and *tblastn*, respectively, with several Elovl sequences from vertebrates as queries. The full-length amino acid (aa) sequences were selected from the sequences retrieved from nr database. Regarding the transcriptomic sequences from TSA database, the sequences containing full-length ORF were selected and translated into deduced aa sequences. After the selection, we divided all Elovl sequences into two groups, namely S/MUFA subfamily (S/MUFA Elovl) and PUFA subfamily (PUFA Elovl) according to Hashimoto *et al*. [4]; S/MUFA Elovl and PUFA Elovl should contain H-W/T-X-H-H and Q/H-X-T/S-X-L-H-X-X-H-H, respectively (electronic supplementary material, table S1). In order to determine orthologues of vertebrate Elovl, human and zebrafish Elovl aa sequences were added to the dataset for the phylogenetic analysis described below. An initial retrieval method of Fads sequences from Echinodermata species was the same as that used for Elovl sequences, although query sequences were several Fads sequences from a sea urchin *P. lividus* and vertebrates. The collected Fads-like sequences were then screened by using three histidine box sequences that are well conserved among Fads (i.e. H-X-X-X-H, H-X-X-H-H and Q-X-X-H-H) (electronic supplementary material, table S1). For the maximum likelihood phylogenetic inference, multiple sequence alignments (MSAs) were created by using MAFFT v7.407 with *einsi* mode [29]. The resulting MSAs were then trimmed by trimAl to remove any columns containing gaps which were >95% of the sequences [30]. After the best-fit aa substitution model was selected as JTT+I+G4+F for S/MUFA Elovl, LG+I+G4 for PUFA Elovl and Fads by ModelTest-NG (v0.1.6) [31], the phylogenetic inferences were performed using RAxML-NG (v1.2.0) with -*all* option (ML search+bootstrapping) [32]. The resulting ML trees were visualized using CLC Main Workbench v21 (Qiagen).

### 2.4. Functional characterization in the yeast *Saccharomyces cerevisiae*

PCR for the full-ORF amplification was performed with PrimeSTAR Max DNA Polymerase (TaKaRa Bio Inc. Shiga, Japan) following the manufacturer’s recommendations. Briefly, a thermocycler condition was set to 98°C for 2 min as the initial denaturation, 35 cycles of 98°C for 10 s, 55°C for 5 s and 72°C for 15 s and then 72°C for 2 min as the final extension. Subsequently, the amplified PCR products were digested using the corresponding restriction enzymes (electronic supplementary material, table S1) and then ligated into similarly digested pYES2 using the LigaFast Rapid Ligation System (Promega Corporation, Madison, WI) following the manufacturer’s recommendations. The ligated product was transformed into *E. coli* using ECOS Competent *E. coli* DH5α (Nippon Gene Co. Ltd, Tokyo, Japan) following the manufacturer’s recommendations. After confirming the sequence (Eurofins Genomics K.K, Tokyo, Japan), the successful plasmid DNA was transformed into the INV*Sc*1 yeast (Thermo Fisher Scientific) using *S.c*. EasyComp™ Transformation Kit (Thermo Fisher Scientific) following the manufacturer’s recommendations. The yeast transformation and culture conditions were described previously [33]. Each transgenic yeast was grown in the presence of one of the potential PUFA substrates for 48 h at 30°C. For Elovl, the exogenously added PUFA substrates were 18:2n-6, 18:3n-3, 18:3n-6, 18:4n-3, 20:4n-6, 20:5n-3, 22:4n-6 and 22:5n-3. We also grew the transgenic yeast without any of the substrate PUFA, only when Elovl had shown activity towards the yeast endogenous SFA and/or MUFA. For Fads, the PUFA substrates were 18:2n-6, 18:3n-3, 20:2n-6, 20:3n-3, 20:3n-6, 20:4n-3, 22:4n-6 and 22:5n-3. In addition to PUFA substrate, 20:1n-7 and 20:1n-9 were also used as the exogenously added substrates for Fads to test Δ5 desaturation activity to produce NMI-FAs. The yeast transformed with empty pYES2 was also cultured as a negative control. The resulting yeast cultures were collected and then lyophilized for the preparation of fatty acid methyl esters (FAME) which were prepared as previously described [33]. All fatty acid substrates were purchased from Nu-Chek Prep Inc. (Elysian, MN), except 18:4n-3 from Matreya LLC (State College, PA) and 20:1n-7, 20:1n-9 and 20:4n-3 from Larodan AB (Solna, Sweden).

### 2.5. Fatty acid analysis by gas chromatography

The FAME prepared from the yeast were analysed by a gas chromatograph (Nexis GC-2030; Shimadzu Corporation, Kyoto, Japan) equipped with a flame ionization detector (FID) and a capillary column (FAMEWAX; 30 m × 0.25 mm i.d. × 0.25 μm, Restek, Bellefonte, PA). The injection port and FID temperatures were set to 250°C and 280°C, respectively. A constant velocity (40 cm s^−1^) was delivered with hydrogen as a carrier gas. The column oven temperatures were programmed for an initial 50°C for 1 min, elevating from 50°C to 190°C at a rate of 40°C min^−1^ and then 190°C to 240°C at a rate of 4°C min^−1^ withholding at a final temperature of 240°C for 3 min. Each detected peak was identified by comparing the retention times of the samples with those obtained from commercial standards. The conversions (%) of each enzyme were calculated according to a formula: [all product areas/(substrate area + all product areas)] × 100.

### 2.6. Structural determination of non-methylene-interrupted fatty acids by gas chromatography–mass spectrometry

To determine the structure of unusual NMI-FAs detected in the yeast assay, we prepared 4,4-dimethyloxazoline (DMOX) derivatives from the FAME samples following a previously described method [11] with a slight modification. Briefly, the FAME samples were transferred into a glass ampoule and 2-amino-2-methyl-1-propanol was added. After closing the ampoule, the mixtures were incubated overnight at 180°C. The resulting DMOX derivatives were extracted by diethyl ether–hexane (1 : 1, v/v), dried and then dissolved in hexane to be injected into a gas chromatography mass spectrometer (GCMS-QP2010; Shimadzu Corporation) equipped with a capillary column (SUPELCOWAX™ 10; 30 m × 0.32 mm i.d. × 0.25 μm, Merck KGaA, Darmstadt, Germany). The injection port and ion source temperatures were 250°C and 200°C, respectively. Helium was used as a carrier gas with a constant velocity (60 cm s^−1^). The programme of column oven temperatures was set to 50°C for 1 min, elevating from 50°C to 180°C at a rate of 40°C min^−1^ and then from 180°C to 240°C at a rate of 1°C min^−1^.

## 3. Results

### 3.1. Sequence and maximum likelihood phylogenetic analysis of *elovl*-like and *fads*-like sequences isolated from *H. pulcherrimus*

**Figure 1.**
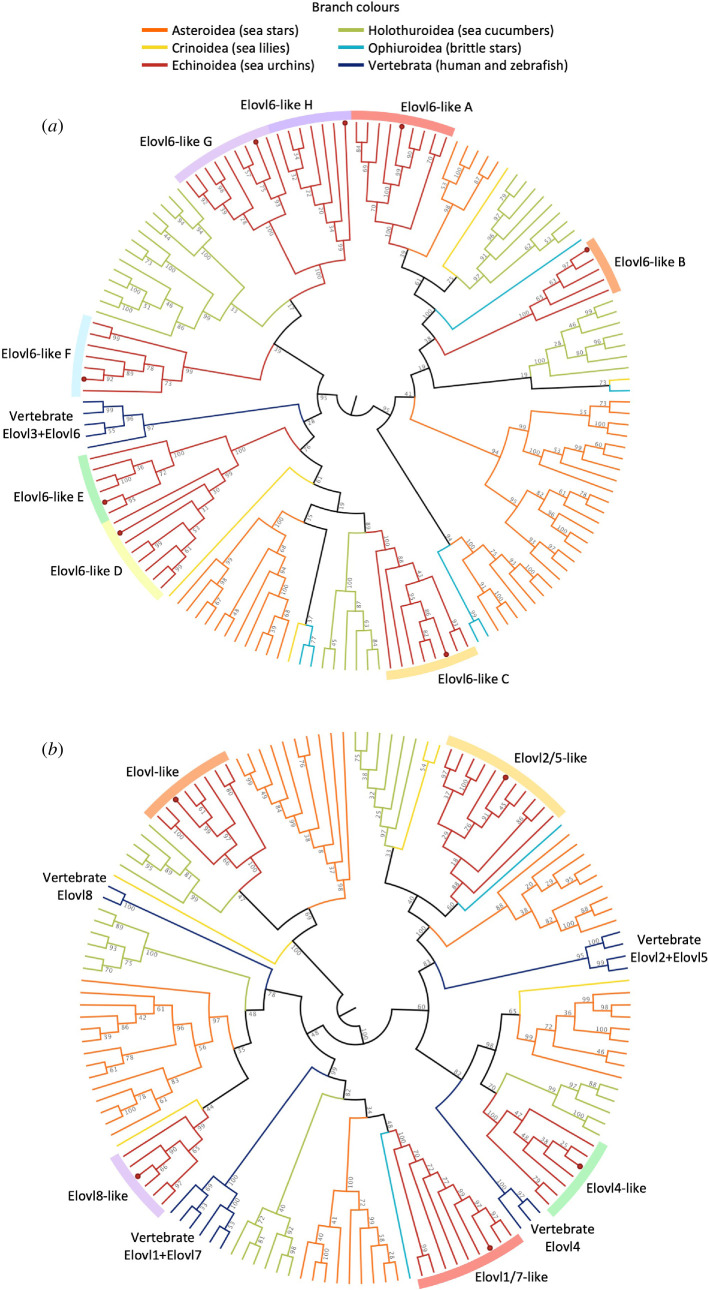
Circular cladograms of the maximum likelihood phylogenetic inference of S/MUFA Elovl (*a*) and PUFA Elovl (*b*) from echinoderm species. *H. pulcherrimus* sequences are indicated by red dots in each leaf node. The fully labelled phylogram trees are provided in electronic supplementary material, figures S2 (S/MUFA Elovl) and S3 (PUFA Elovl).

**Figure 2.**
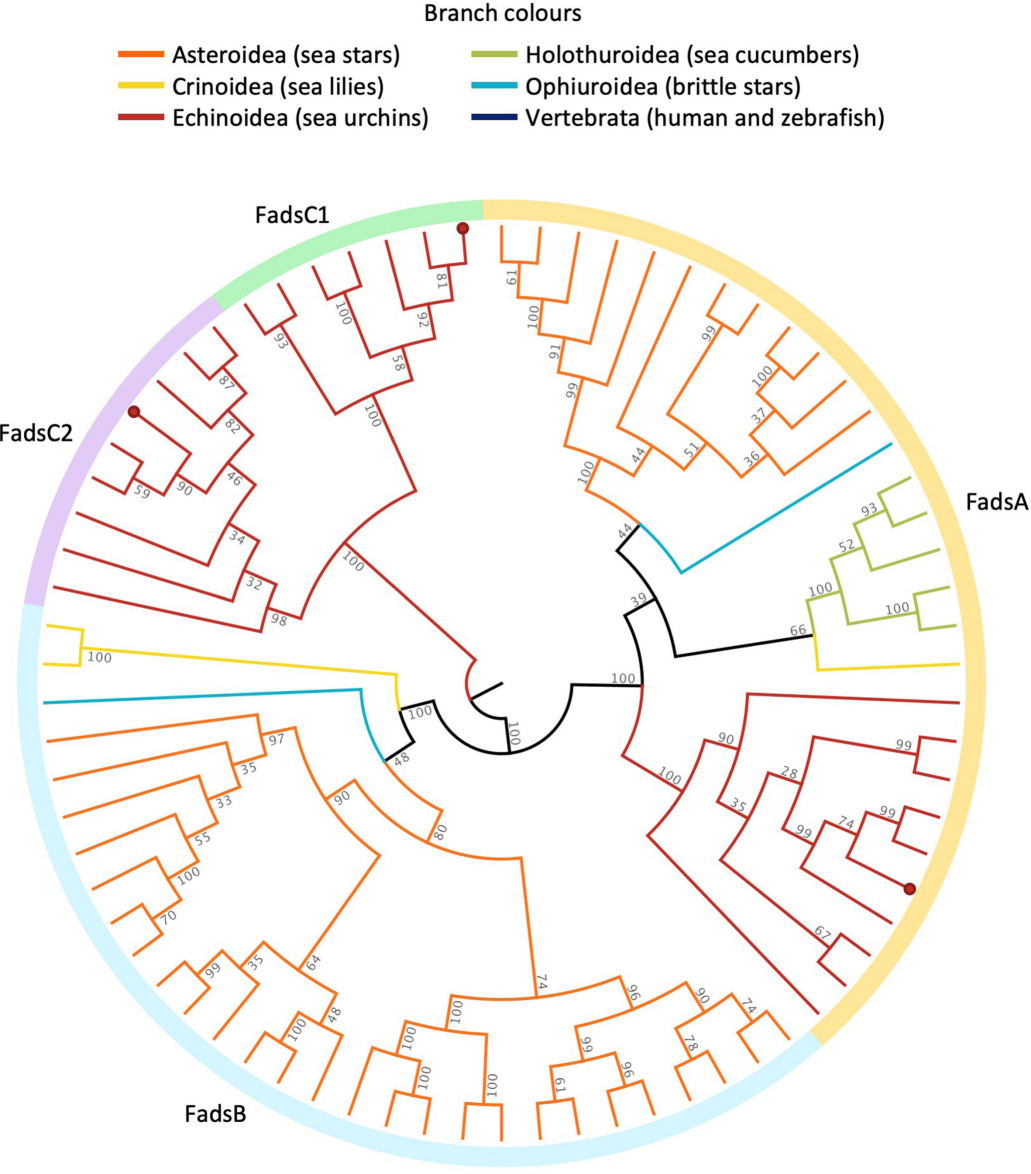
Circular cladogram of the maximum likelihood phylogenetic inference of Fads from echinoderm species. *H. pulcherrimus* sequences are indicated by red dots in each leaf node. The fully labelled phylogram tree is provided in electronic supplementary material, figure S4.

Thirteen *elovl*-like sequences were successfully isolated from *H. pulcherrimus*; 8 and 5 out of 13 were classified as the S/MUFA Elovl and PUFA Elovl, respectively, according to their aa sequences (electronic supplementary material, table S1). Regarding S/MUFA Elovl, we named them as Elovl6-like A–H as Elovl6 is a commonly conserved S/MUFA Elovl among vertebrates (figure 1*a*). Four out of five PUFA Elovl from *H. pulcherrimus* formed relatively well-supported clades with each PUFA Elovl from vertebrate species in the ML phylogenetic tree and, accordingly, we named them Elovl2/5-like, Elovl4-like, Elovl1/7-like and Elovl8-like (figure 1*b*). However, as one PUFA Elovl from *H. pulcherrimus* was not clustered together with any vertebrate Elovl in the ML phylogenetic tree, we named it Elovl-like in the present study. Regarding *fads*, three full-length ORF sequences were successfully isolated from *H. pulcherrimus*. The ML phylogenetic analysis of echinoderm Fads demonstrated that each of *H. pulcherrimus* Fads was clustered together with either FadsA, FadsC1 and FadsC2 from *P. lividus*, and thus the corresponding names were given to each *H. pulcherrimus* Fads (figure 2).

### 3.2. Functional characterization of *H. pulcherrimus* Elovl

**Figure 3.**
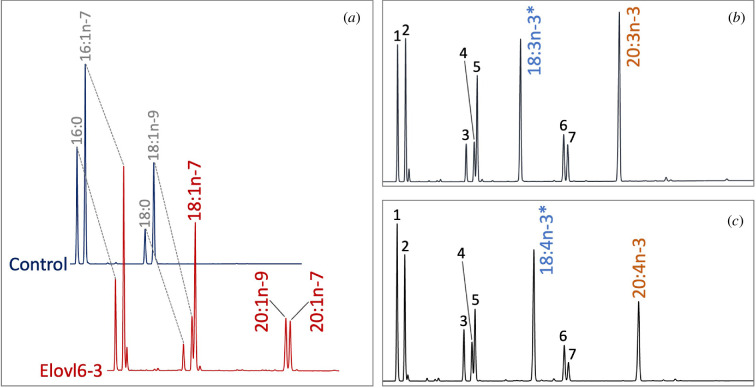
Elovl6-like C isolated from *H. pulcherrimus* showed elongase activities towards various C_18_ MUFA and PUFA to produce the corresponding C_20_ products. (*a*) Gas chromatograms of FAME prepared from the transgenic yeast transformed with an empty pYES2 (control) or pYES2 containing the full-length ORF of *H. pulcherrimus elovl6-like c*. (*b,c*) Gas chromatograms of FAME prepared from the transgenic yeast transformed with pYES2 containing the full-length ORF of *H. pulcherrimus elovl6-like c* and grown in the presence of 18:3n-3 (*b*) and 18:4n-3 (*c*) indicated with an asterisk. Fatty acids are numbered as follows: 16:0 (1), 16:1n-7 (2), 18:0 (3), 18:1n-9 (4), 18:1n-7 (5), 20:1n-9 (6) and 20:1n-7 (7).

Regarding S/MUFA Elovl, 7 out of 8 Elovl showed almost undetectable level of activity towards all substrates tested (table 1). However, surprisingly, along with an elongase capacity towards the yeast endogenous MUFA (i.e. 16:1n-7 and 18:1n-9) to produce 18:1n-7 + 20:1n-7 and 20:1n-9, respectively (figure 3*a*; electronic supplementary material, table S2), prominent conversions towards all C_18_ PUFA substrates were observed in the yeast expressing *elovl6-like C* (figure 3*b*,*c*). Table 2 shows substrate conversions (%) of all PUFA Elovl isolated from *H. pulcherrimus*. All PUFA Elovl possessed an elongation capacity towards a series of C_18_ and C_20_ PUFA substrates to produce corresponding +C_2_ products. However, most of the conversions are overall low (below 5%) except Elovl1/7-like showing clear elongations towards ARA (20:4n-6) and EPA (20:5n-3) (table 2). The Elovl1/7-like further showed an activity towards C_22_ PUFA substrates to produce the corresponding C_24_ products (i.e. 24:4n-6 and 24:5n-3) (table 2).

**Table 1.** Substrate conversion of the yeast *S. cerevisiae* transformed with pYES2 containing the ORF of the *H. pulcherrimus* elongases belonging to S/MUFA subfamily defined by Hashimoto *et al*. [4]. n.d., not detected.

substrate	product	conversion (%)								activity
		Elovl6-like A	Elovl6-like B	Elovl6-like C	Elovl6-like D	Elovl6-like E	Elovl6-like F	Elovl6-like G	Elovl6-like H	
		OR859744	OR859745	OR859746	OR859747	OR859748	OR859749	OR859750	OR859751	
18:2n-6	20:2n-6	0.5	1.5	48.0	1.2	0.7	0.4	3.7	0.7	C_18_→C_20_
18:3n-3	20:3n-3	0.4	2.4	58.5	1.6	1.6	0.5	1.2	2.4	C_18_→C_20_
18:3n-6	20:3n-6	0.4	0.5	39.7	0.8	0.6	0.4	1.2	0.5	C_18_→C_20_
18:4n-3	20:4n-3	0.6	0.9	41.4	1.2	1.0	0.5	0.8	0.6	C_18_→C_20_
20:4n-6	22:4n-6	n.d.	n.d.	1.6	0.1	n.d.	n.d.	0.4	n.d.	C_20_→C_22_
20:5n-3	22:5n-3	n.d.	n.d.	1.4	0.3	0.6	n.d.	0.5	n.d.	C_20_→C_22_
22:4n-6	24:4n-6	n.d.	n.d.	n.d.	n.d.	n.d.	n.d.	n.d.	n.d.	C_22_→C_24_
22:5n-3	24:5n-3	n.d.	n.d.	n.d.	n.d.	n.d.	n.d.	n.d.	n.d.	C_22_→C_24_

**Table 2.** Substrate conversion of the yeast *S. cerevisiae* transformed with pYES2 containing the ORF of the *H. pulcherrimus* elongases belonging to PUFA subfamily defined by Hashimoto *et al*. [4]. n.d., not detected.

substrate	product	conversion (%)					activity
		Elovl2/5-like	Elovl-like	Elovl1/7-like	Elovl4-like	Elovl8-like	
		OR753998	OR753999	OR754000	OR754001	OR754002	
18:2n-6	20:2n-6	1.6	1.1	3.4	0.6	1.5	C_18_→C_20_
18:3n-3	20:3n-3	2.5	0.2	1.9	0.7	3.9	C_18_→C_20_
18:3n-6	20:3n-6	3.4	0.6	8.6	0.7	0.8	C_18_→C_20_
18:4n-3	20:4n-3	5.7	0.4	3.0	0.8	0.7	C_18_→C_20_
20:4n-6	22:4n-6	3.1	0.2	21.7	0.4	2.0	C_20_→C_22_
20:5n-3	22:5n-3	3.7	0.4	11.6	0.9	1.7	C_20_→C_22_
22:4n-6	24:4n-6	n.d.	n.d.	9.5	n.d.	n.d.	C_22_→C_24_
22:5n-3	24:5n-3	n.d.	n.d.	14.7	n.d.	n.d.	C_22_→C_24_

### 3.3. Functional characterization of *H. pulcherrimus* Fads

**Figure 4.**
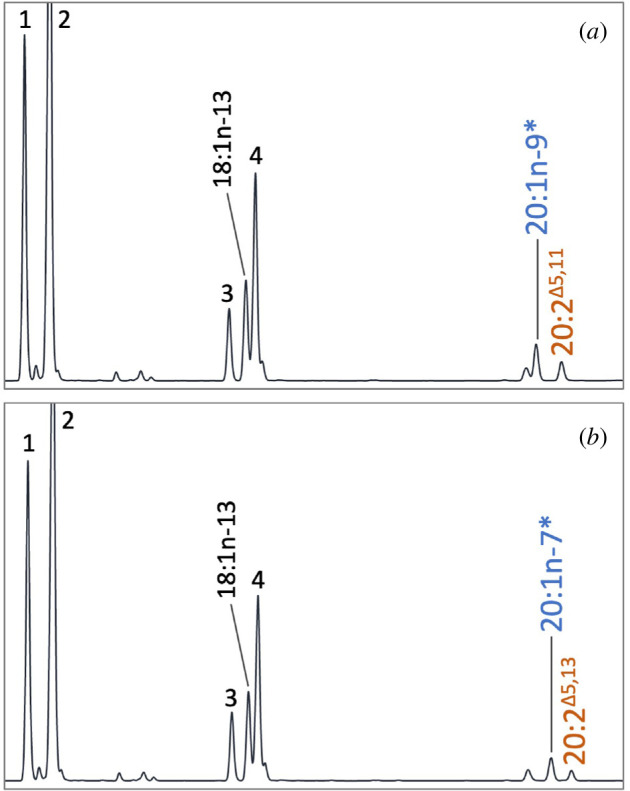
*H. pulcherrimu*s FadsA enables production of C_20_ non-methylene-interrupted dienoic fatty acids. Gas chromatograms of FAME prepared from the transgenic yeast transformed with pYES2 containing the full-length ORF of *H. pulcherrimus fadsa* and grown in the presence of 20:1n-9 (*a*) and 20:1n-7 (*b*) indicated with an asterisk. 18:1n-13 is a Δ5-desaturated product from 18:0. The yeast endogenous fatty acids are numbered as follows: 16:0 (1), 16:1n-7 (2), 18:0 (3), 18:1n-9 (4).

As shown in table 3, clear product peaks (ARA and EPA) were observed in the transgenic yeast expressing *H. pulcherrimus* FadsA grown in the presence of 20:3n-6 and 20:4n-3, respectively, denoting that FadsA is a Δ5 desaturase (table 3). The Δ5 desaturation by FadsA can also happen onto 20:2n-6 and 20:3n-3 to produce NMI-FAs 20:3^Δ5,11,14^ and 20:4^Δ5,11,14,17^, respectively. In addition, the transgenic yeast expressing FadsA was able to desaturate MUFA substrates, namely 20:1n-9 and 20:1n-7 into the NMI-FAs 20:2^Δ5,11^ and 20:2^Δ5,13^, respectively (figure 4, table 4). The mass spectra of DMOX derivative of all those NMI-FAs contained a diagnostic ion for Δ5 desaturation at *m*/*z* = 153 (electronic supplementary material, figures S5 and S6). The 20:2^Δ5,11^ and 20:2^Δ5,13^ were clearly distinguished by the position of the characteristic gaps of 12 atomic mass units (a.m.u.) between *m*/*z* = 222 and 234, and *m*/*z* = 250 and 262, respectively, in their mass spectra (electronic supplementary material, figure S5). The MS profiles of 20:3^Δ5,11,14^ and 20:4^Δ5,11,14,17^ also exhibited the characteristic 12 a.m.u. gaps indicating the double bonds at Δ11,14 and Δ11,14,17, respectively (electronic supplementary material, figure S6). Unlike FadsA, FadsC2 showed clear Δ8 desaturase activity towards 20:2n-6 and 20:3n-3 to produce 20:3n-6 and 20:4n-3, respectively (table 3), while only trace levels of activities towards 20:3n-3 and 20:3n-6 were detected in the yeast expressing FadsC1 (table 3).

**Table 3.** Substrate conversion of the yeast *S. cerevisiae* transformed with pYES2 containing the ORF of the *H. pulcherrimus* fatty acyl desaturase. n.d., not detected.

substrate	product	conversion (%)			activity
		FadsA	FadsC1	FadsC2	
		OR545538	OR545539	OR545540	
18:2n-6	18:3n-6	n.d.	n.d.	0.5	Δ6
18:3n-3	18:4n-3	n.d.	n.d.	0.1	Δ6
20:2n-6	20:3n-6	n.d.	n.d.	40.5	Δ8
20:3n-3	20:4n-3	n.d.	0.2	11.7	Δ8
20:3n-6	20:4n-6	50.2	n.d.	n.d.	Δ5
20:4n-3	20:5n-3	34.7	n.d.	n.d.	Δ5
22:4n-6	22:5n-6	n.d.	n.d.	n.d.	Δ4
22:5n-3	22:6n-3	n.d.	n.d.	n.d.	Δ4

**Table 4.** Δ5 desaturase activity towards various C_20_ substrates to produce NMI-FAs in the transgenic yeast expressing *H. pulcherrimus* FadsA.

substrate	product	conversion (%)	activity
20:1n-9	20:2^∆5,11^	35.1	Δ5
20:1n-7	20:2^Δ5,13^	29.7	Δ5
20:2n-6	20:3^∆5,11,14^	28.9	Δ5
20:3n-3	20:4^∆5,11,14,17^	14.5	Δ5

## 4. Discussion

In marine organisms, LC-PUFA have important roles in several physiological processes such as reproduction [34,35], brain and eye development [36,37] and growth performance [38]. Studies have shown that dietary LC-PUFA can significantly promote growth in juvenile sea urchins, including *P. lividus* [39]. However, other research indicates that sea urchins can achieve normal development and growth without dietary LC-PUFA [22–24]. For example, although ARA enhances gonad development, nutritional value and growth performance in adult *S. intermedius* [40], DHA appears non-essential as its deficiency does not affect gametogenesis or survival [40,41]. The content of DHA in sea urchins remains very low on a DHA-deficient diet, and the absence of ARA in the diet does not affect its levels in sea urchins, when 18:2n-6 was provided in their diet. Similarly, the levels of EPA in sea urchins are unaffected by diets lacking EPA but containing 18:3n-3 [42,43], suggesting their endogenous capability to biosynthesize these LC-PUFA from C_18_ precursors. This was partially confirmed by the presence of functional desaturases with Δ5 and Δ8 desaturase activity in *P. centrotus* [11]. Moreover, several studies have shown that the expression levels of genes encoding Fads and/or Elovl in the gonad and digestive tract in *S. intermedius* are significantly affected by dietary LC-PUFA compositions, highlighting the role of these genes in LC-PUFA biosynthesis and adaptation to dietary LC-PUFA availability, with the gonad and digestive tract serving as important sites for this process [14–16,18].

**Figure 5.**
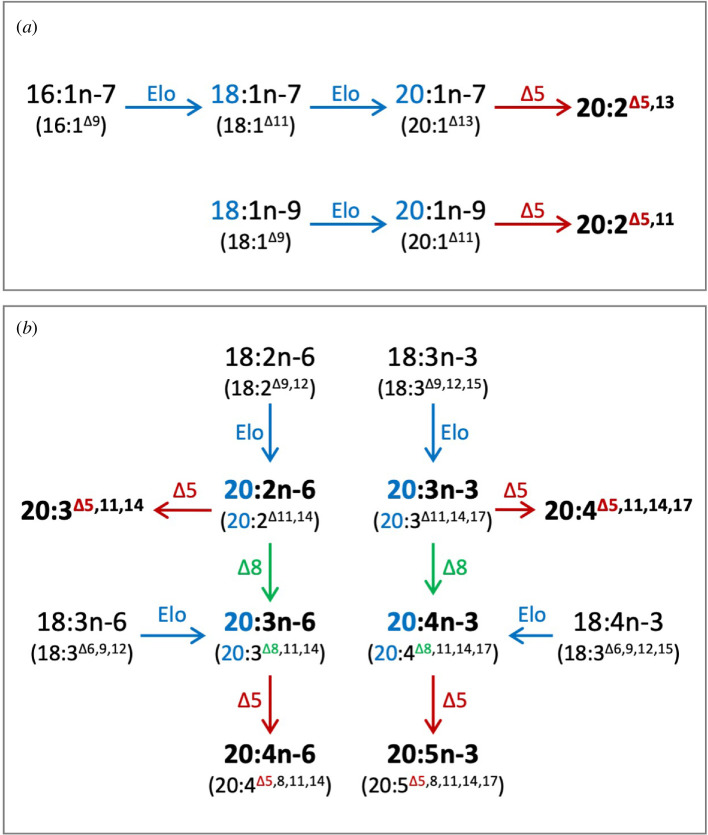
Biosynthetic pathways of various C_20_ PUFA in *H. pulcherrimus*. (*a*) The biosynthetic pathways of C_20_ dienoic NMI-FAs from C_16_ and C_18_ MUFA substrates. (*b*) The biosynthetic pathways of various C_20_ PUFA including physiologically important ARA (20:4n-6) and EPA (20:5n-3) as well as trienoic and tetraenoic NMI-FAs (20:3^Δ5,11,14^ and 20:4^Δ5,11,14,17^, respectively).

The present study unequivocally demonstrates that the sea urchin *H. pulcherrimus* possesses a complete set of enzymes necessary to synthesize ARA and EPA endogenously from their corresponding C_18_ PUFA precursors, 18:2n-6 and 18:3n-3, respectively. This biosynthesis occurs via the ‘Δ8 pathway’, which consists of a sequence of elongation, followed by Δ8 desaturation and then Δ5 desaturation (electronic supplementary material, figure S1) [44,45]. Unlike vertebrates such as mammals and teleosts, which possess Fads with Δ6 desaturase activity (e.g. Fads2) that enable the operation of the ‘Δ6 pathway’, *H. pulcherrimus* lacks Fads with this activity. As a result, this species is unable to use the ‘Δ6 pathway’, which typically involves Δ6 desaturation, elongation and Δ5 desaturation (electronic supplementary material, figure S1). Nonetheless, this finding underlines the importance of including these C_18_ precursors in the sea urchin diet to facilitate the synthesis of ARA and EPA. Furthermore, the presence of multiple copies of potential orthologues of vertebrate Elovl6, characterized as a MUFA elongase, were found in *H. pulcherrimus*. One of these, Elovl6-like C, appears to play a prominent role in LC-PUFA biosynthesis. Functional analysis of these enzymes also allowed us to delineate C_20_ NMI-FA biosynthetic pathways in *H. pulcherrimus*. This analysis demonstrated that, through the action of enzymes isolated in the present study, including FadsA with Δ5 desaturase activity and several elongases like Elovl6-like C, *H. pulcherrimus* is capable of synthesizing a range of C_20_ methylene-interrupted PUFA (20:2n-6, 20:3n-3, 20:3n-6, 20:4n-3, ARA and EPA) and four C_20_ NMI-FAs (20:2^Δ5,11^, 20:2^Δ5,13^, 20:3^Δ5,11,14^ and 20:4^Δ5,11,14,17^) from C_18_ precursors (figure 5).

The comprehensive retrieval of Elovl enzymes revealed the presence of numerous elongases in echinoderms, consistent with the previous findings reported by Liu *et al*. [19]. A particularly notable finding is the marked increase in the copy number of *elovl6-like* gene in sea urchin species. In the ML phylogenetic analysis, each sea urchin Elovl6-like (A–H) formed a well-supported clade (figure 1*a*), suggesting similar PUFA elongase activity across these variants as observed in *H. pulcherrimu*s. However, the phylogenetic tree resolution was not sufficiently high to fully determine the presence, absence and relationships of each Elovl6-like orthologue among different echinoderms. Interestingly, a clade containing several sea cucumber sequences with unknown function appeared to be a direct sister group to the sea urchin Elovl6-like C. Given the close relation between sea urchins and sea cucumbers [46,47], it is likely that *elovl6-like C* gene emerged in their common ancestor. Although mammalian Elovl6 has been characterized as an elongase showing activity towards SFA and MUFA, none of the Elovl6 in *H. pulcherrimus* exhibited an elongase activity towards the yeast endogenous MUFA (16:1n-7 and 18:1n-9) except for Elovl6-like C. This enzyme was capable of elongating several C_18_ unsaturated fatty acid substrates regardless of their degree of unsaturation, making it potentially the most physiologically significant among the Elovl6-like enzymes isolated. A similar pattern has been observed in crustaceans such as the orange mud crab *Scylla olivacea* and the purple land crab *Gecarcoidea lalandii*, where Elovl6 shows elongase activity not only towards S/MUFA but also towards various PUFA substrates [48,49]. Additionally, Elovl6 from the gammarid amphipod *Echinogammarus marinus* displayed some ability to elongate C_18_ PUFA substrates [50]. These findings suggest a potential functional diversification in substrate preferences among invertebrate Elovl6.

Regarding PUFA elongases, four Elovl from *H. pulcherrimus* (Elovl2/5-like, Elovl4-like, Elovl1/7-like and Elovl8-like) clustered with corresponding vertebrate Elovl enzymes in phylogenetic analysis (figure 1*b*). However, there were no exact one-to-one orthologous enzymes for Elovl2 and Elovl5, which are thought to be the main enzymes involved in LC-PUFA biosynthesis in vertebrates, as well as Elovl1 and Elovl7, likely owing to gene duplications in ancestral vertebrates or later-diverging deuterostomes [51]. Unlike these four, one Elovl from *H. pulcherrimus* did not cluster with any vertebrate Elovl and is likely widespread among echinoderm species, including sea stars, sea cucumbers and one sea lily. Most Elovl showed elongase activity towards multiple C_18_ and C_20_ PUFA substrates, but only Elovl1/7-like was capable of elongating C_22_ PUFA substrates to produce C_24_ products (24:4n-6 and 24:5n-3). Although elongase activity towards C_22_ PUFA substrates has not been tested in mammalian ELOVL1 and ELOVL7 [52,53], Elovl1/7 (also referred to as Elovl7 or Elovl1 in some studies) from certain crustaceans has shown elongase activity towards C_22_ PUFA substrates [49,50,54,55]. However, to the best of our knowledge, C_24_ PUFA products have not been reported in sea urchin tissues, suggesting limited biological significance. Indeed, another C_24_ PUFA, 24:6n-3, is uncommon in sea urchin species, albeit the presence of a certain level of C_24_ PUFA in some other echinoderms (brittle stars and sea lilies) [8]. Furthermore, as none of the *H. pulcherrimus* Fads showed Δ6 desaturase activity towards 24:5n-3, DHA biosynthesis via the so-called ‘Sprecher pathway’ cannot occur in this species.

The regioselectivities detected in three Fads from *H. pulcherrimus* are fairly consistent with those of previous studies in *P. lividus* [11]; one FadsA with a Δ5 desaturase activity and two FadsC with either overall low activity (FadsC1) or a Δ8 desaturase activity (FadsC2). In addition to the previously reported Δ5 desaturase activity towards 20:2n-6 and 20:3n-3 to produce NMI-FAs 20:3^Δ5,11,14^ and 20:4^Δ5,11,14,17^, respectively [11], FadsA from *H. pulcherrimus* was able to introduce Δ5 desaturation in two MUFA substrates 20:1n-9 and 20:1n-7 to produce 20:2^Δ5,11^ and 20:2^Δ5,13^, respectively. Because sea urchins generally contain significant levels of those dienoic NMI-FAs [8], this activity would be a common feature of FadsA in sea urchins. Furthermore, given that the FadsA was widespread among all echinoderm classes and Δ5-desaturated NMI-FAs are commonly found in not only sea urchins but also other echinoderms such as sea cucumbers and sea stars [8,11], it could be speculated that those Δ5-desaturated NMI-FAs were endogenously synthesized and accumulated in their bodies, despite unknown physiological functions. Unlike FadsA, distributions of FadsB and FadsC were more class-specific; FadsB is present in sea star, brittle star and sea lily species, and FadsC (FadsC1 and FadsC2) are present exclusively in sea urchin species. Considering the phylogenetic relationships of echinoderms [46,47], *fadsb* gene might be lost in the common ancestor of Echinozoa (sea urchins and sea cucumbers).

In conclusion, the present study established that *H. pulcherrimus* possessed 13 Elovl and three Fads enzymes potentially involved in PUFA biosynthesis. Notably, Elovl6-like C exhibits elongase activity towards C_18_ PUFA substrates to produce several C_20_ PUFA products, an unexpected function given that Elovl6 is typically categorized as a S/MUFA elongase in vertebrate species. This finding underscores the risk of drawing conclusions about enzymatic functions based solely on aa sequences and/or phylogenetic analysis, which could lead to inaccurate interpretations of a species’ biosynthetic capabilities of PUFA. Regarding desaturase functions, Δ5 desaturase (FadsA) and Δ8 desaturase (FadsC2) activities were found in *H. pulcherrimus*. Together with Elovl activities, *H. pulcherrimus* is capable of synthesizing physiologically important ARA and EPA from 18:2n-6 and 18:3n-3, respectively, via the so-called ‘Δ8 pathway’ [11]. In addition, FadsA was able to synthesize several NMI-FAs from both C_20_ PUFA and MUFA substrates. The results obtained in the present study provide solid molecular evidence for the dietary essential fatty acids in the sea urchin and the endogenous production of particularly unusual fatty acids, NMI-FAs.

## Data Availability

The full-length ORF sequences isolated from *H. pulcherrimus* in the present study were deposited into the NCBI GenBank; S/MUFA elongases (accessions OR859744–OR859751), PUFA elongases (OR753998–OR754002) and fatty acid desaturases (OR545538–OR545540). Our data are provided as supplementary figures and tables [56].
